# Effect of the Class C Fly Ash on Low-Reactive Gold Mine Tailing Geopolymers

**DOI:** 10.3390/polym14142809

**Published:** 2022-07-09

**Authors:** Yibran Perera-Mercado, Ahmadreza Hedayat, Lori Tunstall, Cara Clements, Julia Hylton, Linda Figueroa, Nan Zhang, Héctor Gelber Bolaños Sosa, Néstor Tupa, Isaac Yanqui Morales, Reynaldo Sabino Canahua Loza

**Affiliations:** 1 Department of Civil and Environmental Engineering, Colorado School of Mines, 1500 Illinois St., Golden, CO 80401, USA; hedayat@mines.edu (A.H.); ltunstall@mines.edu (L.T.); caraclements@mines.edu (C.C.); juliahylton@mines.edu (J.H.); lfiguero@mines.edu (L.F.); zhangnan@mines.edu (N.Z.); 2 Department of Metallurgy Engineering, Universidad Nacional de San Agustín de Arequipa, Arequipa 04000, Peru; hbolanos@unsa.edu.pe (H.G.B.S.); ntupaf@unsa.edu.pe (N.T.); iyanqui@unsa.edu.pe (I.Y.M.); rcanahua@unsa.edu.pe (R.S.C.L.)

**Keywords:** mine tailings, class C fly ash, geopolymers, Rietveld method

## Abstract

Beneficiation of industrial wastes, such as mine tailings (MTs), through development of alternative eco-friendly geopolymer binders for construction composites offers a twofold environmental benefit, as it reduces the demand for cement and it increases the sustainability of industrial processes by creating a value-added product from an industrial byproduct. While MTs have the requisite composition for use as a geopolymer precursor, they are often low-reactive. This study explored the effect of Class C Fly Ash (FAc) on the geopolymerization of low-reactive gold MTs. A 10 M sodium hydroxide (NaOH) solution was used as the alkaline activator with four different concentrations of FAc (5, 10, 15 and 20 wt.%). The results indicated that the combination of FAc with the low-reactive gold MTs improved the physicochemical stability of the final geopolymerized samples, with a 95–120% increase in compressive strength, compared to the geopolymer samples of only low-reactive gold MTs. Although some of the strength improvement could be attributed to geopolymerization of the FAc itself, the presence of the FAc also improved the reactivity of the MTs, increasing the geopolymer production of the MTs. This study documents the positive effects of the FAc on gold MTs with low-calcium content and their conversion into sustainable inorganic composite geopolymers for the construction field.

## 1. Introduction

The solid waste generated during the extraction of gold by mining and mineral processing activities are known as gold mine tailings (MTs). This type of waste can cause severe damage to the environment during stockpiling as a result of the leaching of metals and processing chemicals into the ecosystem. Due to the difficulty of managing these solid wastes, several studies have been carried out to reuse MTs as raw materials in the construction field; however, managing leaching from these solid wastes while providing physical durability has been an ongoing challenge [1,2,3,4,5]. One promising technique is to utilize the waste stream for the development of alternative eco-friendly materials, such as geopolymer-based systems [2,3,6,7,8].

Geopolymers are strong and durable cementitious materials that result from the alkali activation of aluminosilicates. Commonly, the aluminosilicate source materials either occur naturally (e.g., kaolin, metakaolin, rice husk ash, volcanic rock powders, etc.) or are produced by industrial processes (e.g., fly ash, blast furnace slag, mine tailings (MTs), etc.) [3,4,5,6,7,8,9]. The chemical composition, mineralogical structure, fineness, and amorphous content of these materials affect the reactivity of the aluminosilicate sources [2,6,10,11]. Some MTs can be stabilized or solidified through alkaline activation to create a cementitious material through the inorganic polymerization of their compounds, explained as follows. The geopolymer network consists of a porous structure formed by silicate (SiO_4_) and aluminate (AlO_4_) tetrahedron units and includes an Si-O-Al linkage in three directions by sharing the oxygen atoms between the tetrahedral units. The empirical formula of the geopolymer structure is shown in Equation (1),

M_n_^+^[-(SiO_2_)_z_-AlO_2_-]_n_ (1)

where M^+^ is an alkali cation, such as sodium (Na^+^), potassium (K^+^), or calcium (Ca^+^); z is the Si/Al ratio; and n is the degree of polymerization [8].

On the other hand, fly ash is also an industrial byproduct, generated in coal combustion plants, and is routinely used as an aluminosilicate source material in geopolymerization. Fly ash is a relatively common solid waste and has advantages in terms of particle size, distribution, and reactivity [2], particularly if it is a high-calcium fly ash (like class C fly ash), which is more reactive for applications in the building materials field. Geopolymers containing a low percentage of calcium in the precursor materials are primarily composed of a sodium aluminosilicate hydrate (N-A-S-H) product, whereas high calcium content results in the formation of a calcium aluminosilicate hydrate (C-A-S-H) binder, which is most similar to the hydration product produced by Portland cement, the C-S-H gel [3,12].

Several researchers report that gold MTs have low reactivity, and require the addition of a supplementary material to achieve a strong matrix by alkali activation [4,5,13]. The work presented in this study focused on utilizing gold MTs as the primary source material and fly ash (FAc) as a supplementary material and explored the effect of FAc concentration on the geopolymerization of low-reactive gold MTs for the generation of inorganic co-geopolymerized composite materials.

## 2. Materials and Methods

### 2.1. Raw Materials Characterization

**Figure 1:**
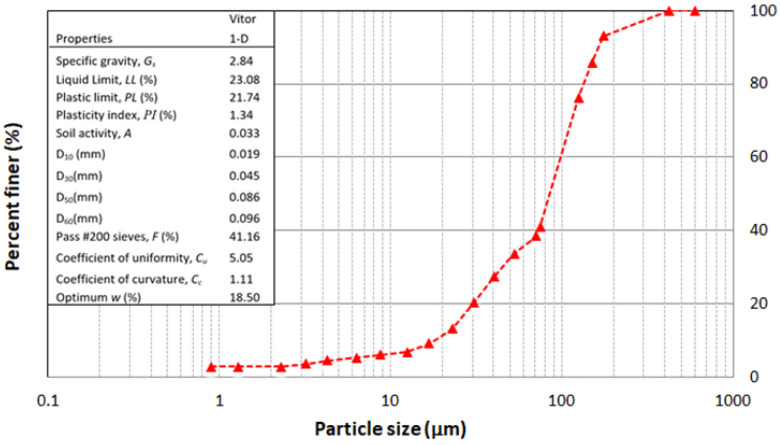
Particle size distribution of the raw MTs.

Gold MTs sampled from Vitor (Arequipa, Perú) were used in this study. The geotechnical characterizations were performed following ASTM standards D6913 [14], D7928 [15], and D4318 [16]. The grain size distributions of the raw materials are shown in Figure 1. The embedded table in Figure 1 lists the parameters: (1) the mean particle size D50 = 0.086 mm with fines percentage equal to 41% and (2) a coefficient of uniformity Cu = 5. The MTs were classified as silty sand (SS) with low plasticity according to USCS classification standards, having a low liquid limit (23%), low plasticity (PI = 1.3%), and low capacity for holding water (A = 0.033). On the other hand, the fly ash used in this study (provided by Holcim in Denver, Colorado, US.) contained about 24 wt.% calcium oxide, and, thus, was classified as Class C, according to ASTM C618-19 [17,18].

### 2.2. Production of Geopolymer Specimens

**Figure 2:**
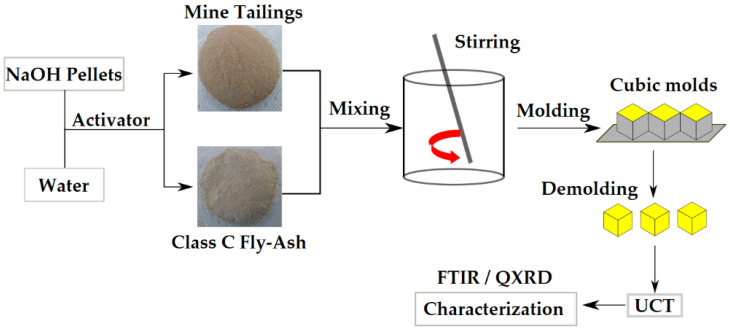
Geopolymer samples production process. Reprinted from Ref. [19].

Prior to mixing, the raw MTs were manually crushed using a hammer, and sieved to a particle size below 600 μm. The size reduced MTs were dry mixed in different proportions with 5, 10, 15 and 20 wt.% of FAc. The geopolymer pastes were then made by hand combining the mixture of raw MTs and FAc with a 10 M NaOH solution until a uniform final paste was obtained. The water/mass ratio used for all GP specimens were 0.18. The NaOH solution was prepared by stirring water with NaOH pellets at 97% purity (Sigma-Aldrich) [19,20]; and the solutions were then placed at room temperature (21 ± 1 °C) for 30 min to dissipate the heat generated during dissolution. The pastes were placed in cubic molds of 5 × 5 × 5 cm^3^ and compacted into three different layers with a Harvard miniature compaction (HMC) tamper. Each layer was tamped 71 times, which was calculated as equivalent to the HMC mold, such that the numbers per unit cross-sectional area were constant. The molds were primed with a WD-40 lubricant to facilitate release of the samples from the molds. The schematic production process of the geopolymer samples is shown in Figure 2 [19].

The cubic samples were demolded, put in containers, and covered with plastic wrapping to avoid water evaporation during the curing step and then placed in an oven with a temperature of 40 ± 1 °C for 24 h to prevent the cracking due to the thermal shock. Then, the oven temperature was increased to 70 ± 1 °C for three days [13,19]. After four days of curing, the plastic wrapping was removed, and the specimens were placed in the oven for another three days for drying at the same temperature of 70 ± 1 °C. A total of three cubic specimens for each system in the study were cast for compression testing and these same samples were used for further materials characterization. Two control systems were also produced pure geopolymers specimens based only on each individual precursor (original GP.-MTs, and FAc GP.) under the same methodology here exposed.

### 2.3. Characterization Techniques

#### 2.3.1. Quantitative X-ray Diffraction (QXRD)

Measurements were made using a Malvern Panalytical X’Pert PRO MPD X-ray diffraction system. The X-ray radiation was Cu Kα, λ = 1.5418 Å and the current was 40 mA with a tension of 45 kV. The scan time and instrument parameters were identical for all the samples with a 2θ angle range between 10° and 90°. The internal standard material added was 9.1 wt.% of Synthetic Diamond [21,22]. The samples were prepared by inter-grinding the samples with the synthetic diamond, using a porcelain mortar.

To identify the crystalline phases using QXRD analysis, the Panalytical X’Pert HighScore software with Rietveld refinement was used [23,24,25]. Although QXRD could only identify crystalline phases, since the wt.% of the internal standard was known, the amorphous content could be determined by normalizing the results to this known 9.1 wt.% of synthetic diamond. The powder diffraction file (PDF) codes (International Centre for Diffraction Data—ICDD) were used to identify the crystalline phases that are given in Table 1.

#### 2.3.2. Fourier Transform Infrared (FTIR)

A Thermo Electron Nicolet 4700 Fourier transform infrared (FTIR) spectrometer was used to collect the spectra in the transmittance mode at specific frequencies ranging from 4000 to 400 cm^−1^. The resulting spectrum analyses were interpreted to identify the specific functional groups within the analyzed samples.

#### 2.3.3. Uniaxial Compressive Tests

The compressive strength of the reacted MT samples was tested following ASTM C109. The MTS Landmark 370.10 machine at a constant displacement rate of 0.21 mm/min was used to evaluate the different geopolymer systems [19,26]. The geopolymer cubes with seven days aging were first placed between two metallic plates to assure that the surfaces were sufficiently flat for the compression test. A total of three measurements (3 cubic samples) were taken and averaged.

#### 2.3.4. Scanning Electron Microscopy/Energy Dispersive Spectroscopy (SEM/EDS)

Both precursors were characterized using a FEI QUANTA 600i Environmental SEM which uses a tungsten filament and is equipped with an EDAX Element SDD EDS detector which was used for the semiquantitative chemical composition average of ten measurements from each specimen.

## 3. Results and Discussion

**Figure 3:**
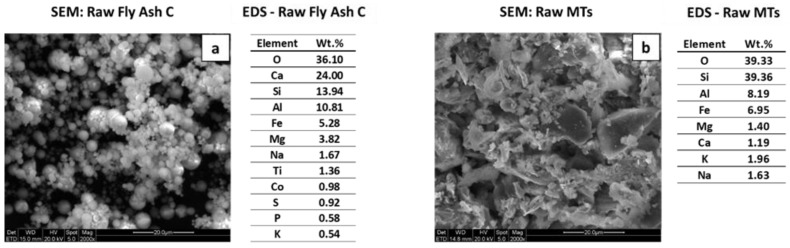
SEM and EDS semiquantitative chemical analyses of the precursor materials (wt.%). (**a**) raw FAc, (**b**) raw MTs.

SEM micrographs of the FAc and gold MTs, and their associated EDS chemical composition analyses, are shown in Figure 3. As expected, the particles of the FAc were spherical in shape with distinct particle sizes (Figure 3a). The EDS semiquantitative chemical composition of the FAc indicated that Ca, Si, Al, Fe, Mg, Na and Ti were the most abundant elements present in the raw FAc material, and Co, P, S, K were less than 1 wt.%. In contrast, the original mine tailings (raw MTs) were composed of angular particles, with finer particles attached to the surface of the coarse particles (Figure 3b). The EDS semiquantitative chemical composition indicated that Si was the primary element of the gold MTs, Al and Fe were secondary elements, and Mg, Ca, K, and Na were present in minor quantities.

**Figure 4:**
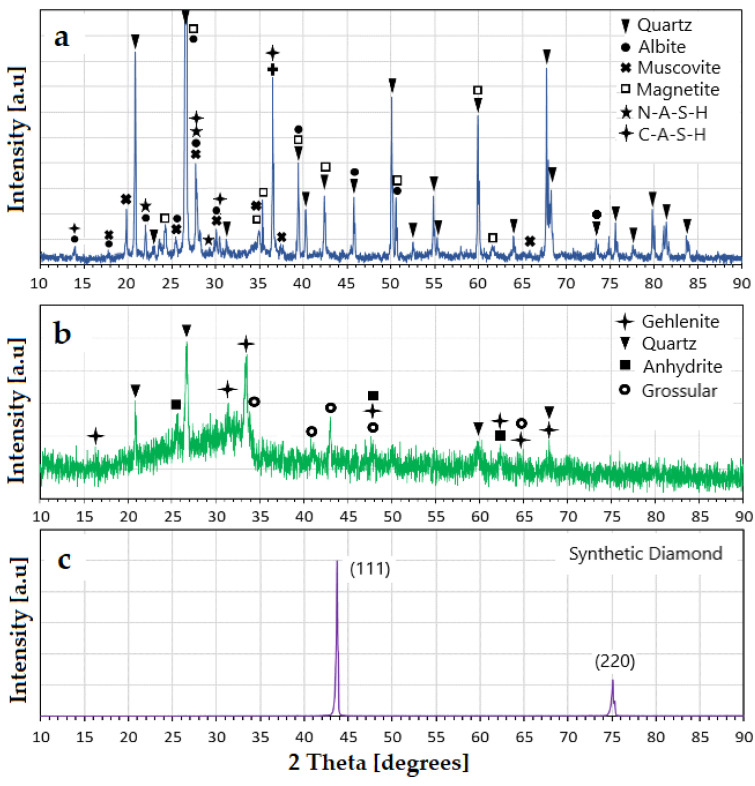
XRD patterns. (**a**) Raw MTs. (**b**) Raw FAc. (**c**) Synthetic diamond used in QXRD analysis.

The X-ray diffraction patterns from both raw minerals used in this research are shown in Figure 4. Synthetic diamond with particle sizes between 2–4 μm was used as an internal standard for this analysis, since its diffraction pattern (shown in Figure 4) did not significantly overlap with the main peaks of the raw MTs and FAc [19]. QXRD analysis revealed that the FAc was about 70.9% amorphous, with remaining crystalline phases of quartz [11,20,27], gehlenite, grossular, and anhydrite.

The raw MTs were primarily a complex mix of crystalline phases (amorphous content of 23.2%), with the primary phase of quartz. Quartz is less reactive than the other aluminosilicate constituents during the alkali activation processes; however, it provides some chemical reactivity in an alkaline environment, and once finely ground, can provide nucleation sites for dispersed formation of hydration products [28,29]. In addition, other phases, such as muscovite [20], magnetite [27,30,31], and albite [27,29] were present in very low percentage. Sodium aluminum silicate hydrate (N-A-S-H) [28], and calcium aluminum silicate hydrate (C-A-S-H) [28] were also identified. The presence of these two hydrated phases could be due to the reaction of the gold MTs that were stored in open air conditions and exposed to different environmental conditions.

**Figure 5:**
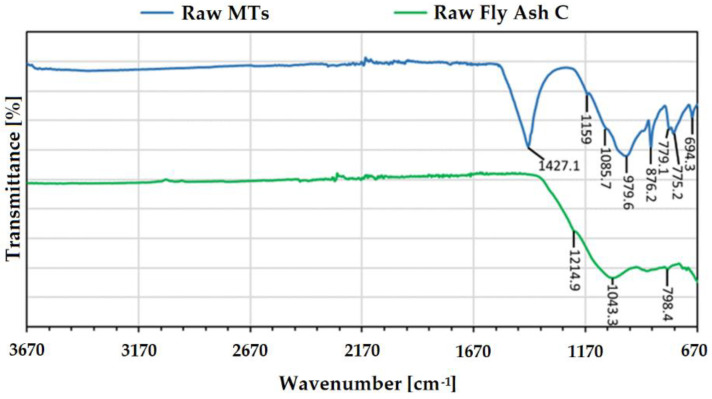
FTIR spectra of the raw FAc and the raw MTs used in this research.

The FTIR analyses from both raw materials are shown in Figure 5. The FTIR spectra from the raw MTs confirmed the presence of quartz, as shown by the characteristic peaks at 1085.7 cm^−1^, and the strong shoulders at 1159 cm^−1^ [9]. The group of signals at 779.1 cm^−1^ and 775.2 cm^−1^ indicated the existence of Si-O-X bonds where X stands for silicates or aluminates [10]. More specifically, the signal at 694.3 cm^−1^ corresponded to the Al-OH bending vibration [32]. The overlapping of signals in the range of 1200–900 cm^−1^ and the presence of a large absorption band indicated aluminosilicate structures with an asymmetric stretching vibration of the Si-O-Si and Si-O-Al bonds in [SiO_4_]^4−^ and [AlO_4_]^5−^ [7,32]. The large absorption band at 1427.1 cm^−1^ indicated the presence of C-O bonds [26,33], and the asymmetric stretching of CO_3_^2−^ was associated with carbonates. In addition, the signal at 876.2 cm^−1^ was also an out-of-plane bending vibration, characteristic to the carbonates [32]. Together, these results indicated the presence of aluminosilicate phases in the raw MTs, corroborated by the previous XRD results.

The FAc spectra show a vibration at 798.4 cm^−1^, associated with the Si-O-Si inter tetrahedral bridging bonds in quartz [32], and the signals at 1214.9 cm^−1^ and 1043.3 cm^−1^ correspond to asymmetric stretching vibration of the Si-O-Si and Si-O-Al bonds in [SiO_4_]^4−^ and [AlO_4_]^5−^ observed in aluminosilicate minerals [32,33], as previously mentioned.

**Figure 6:**
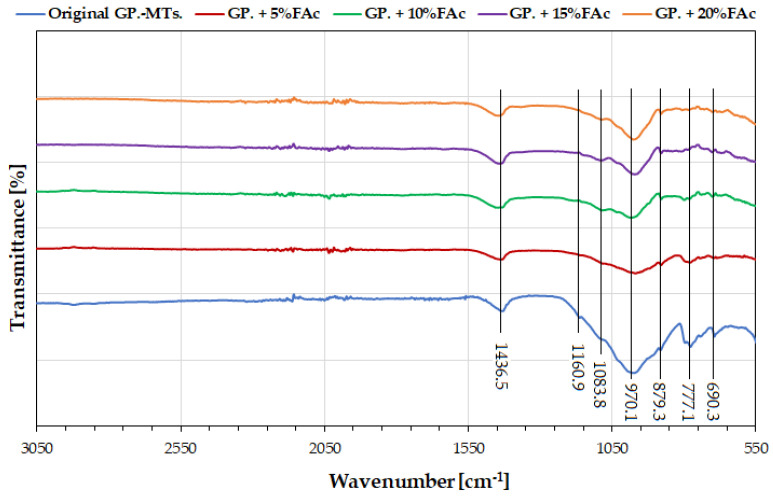
FTIR spectra of the original GP.-MTs, and the supplemented geopolymers with FAc.

On the other hand, Figure 6 shows the FTIR spectra in the region of 3050–550 cm^−1^ from the co-geopolymerized samples (i.e., samples made with both raw MTs and FAc). The band at about 1436.5 cm^−1^ was attributed to the stretching vibrations of the C-O bond of the carbonates (sodium carbonate and calcium carbonate) that appear in all the spectra [12,19,32,33]. A small protrusion at 879.3 cm^−1^, which is an out of plane bending of the carbonate group, was also identified [4,34].

The overlapping of signals in the range of 1200–900 cm^−1^ indicated aluminosilicate structures with an asymmetric stretching vibration of the Si-O-Si and Si-O-Al bonds in [SiO_4_]^4−^ and [AlO_4_]^5−^, which was also seen in the raw MTs (Figure 5). The most distinctive difference between the raw materials (raw MTs and FAc) and the geopolymerized systems was that the main band of the raw MTs (979.6 cm^−1^ in Figure 5) shifted to a lower wavenumber of ~970.1 cm^−1^ after the geopolymerization reaction. According to Yong-Sing et al. [18], this observation indicated the development of amorphous aluminosilicate gel phases, which are expected products of the geopolymerization process [18,35].

As the concentration of FAc increased, absorption bands at 777.1 cm^−1^ and 690.3 cm^−1^ disappeared. Both these bands are associated with silicates or aluminates in the raw MTs [20,32]. Therefore, persistence of these bands in the MT geopolymer (original GP.-MTs) was evidence that only a small degree of geopolymerization had occurred [20,35], while disappearance of these bands with the addition of FAc suggested that the FAc was increasing the dissolution of the MTs.

Table 2 shows the QXRD results of the raw materials (MTs and FAc), the control MTs geopolymer and FAc geopolymer, and the four co-geopolymerized systems at the different MTs/FAc ratios studied in this work. Based on the QXRD results (Table 2), both the raw MTs and the original GP.-MTs contained mainly quartz, muscovite, and albite as primary phases. The original GP.-MTs sample showed the production of an additional phase, zeolite. The presence of the zeolite phase was expected due to the experimental curing parameters used in the production of the geopolymer specimens (pH, temperature, and humidity), and the presence of the N-A-S-H phase in the raw MTs which is known as the zeolitic precursor [36,37].

There was also a reduction in the primary crystalline phases of the original GP.-MTs geopolymer system compared to the raw MTs, indicating that the alkali activation process was successful in dissolving the original crystalline phases for the production of new amorphous phases. The amorphous content in the original GP.-MTs system increased by 19.9 wt.%, compared to the amorphous content of the raw MTs.

A reduction in crystalline content of the gehlenite, grossular, anhydrite and quartz phases were also observed when the raw FAc precursor turned into the FAc geopolymer specimen (Fly Ash C.- GP.), which meant that these crystalline phases were contributing to the generation of binder products during the inorganic geopolymerization step [38,39]. This was further confirmed by changes in the amorphous content, which increased from 70.9% to 95.3% after geopolymerization, a 24.4% increase compared to the raw FAc precursor.

**Figure 7:**
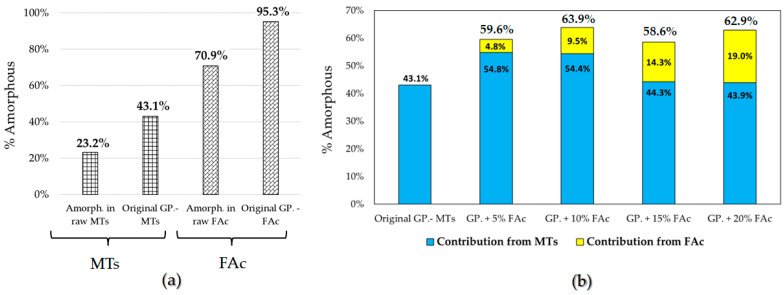
Percentage of amorphous phases calculated QXRD analysis. (**a**) Amorphous content in the raw precursors (raw MTs, raw FAc) and in their individual geopolymer systems. (**b**) content in the original GP.-MTs, and contribution of amorphous content from each precursor in the co-geopolymerized samples.

In the co-geopolymerized samples (GP. + x% FAc), there appeared to be a synergistic effect between the MT and FAc that promoted the dissolution of the crystalline phases in the raw materials and the generation of additional amorphous content (Figure 7) with a low quantity of the formed C-A-S-H phase in some of the geopolymerized specimens [40,41]. Alone, the original GP.-MTs sample had an amorphous content of 43.1 wt.%. However, with just a small amount of FAc (5 wt.%), the amorphous content of the co-geopolymerized sample increased from 43.1 wt.% to 59.6 wt.% (GP. + 5% FAc), a difference of 16.5 wt.%. Since the FAc-GP. could only produce a maximum of 95.3 wt.% amorphous content (Figure 7a), the change in amorphous content in the co-geopolymerized sample cannot be attributed to additional amorphous content from the FAc alone (Figure 7b). In other words, if we assume that the FAc portion of the co-geopolymerized sample still became 95.3 wt.% amorphous after geopolymerization, its total contribution to the amorphous content of the co-geopolymerized sample could only be 4.8 wt.% (0.05 × 95.3), since the FAc was only 5 wt.% of the sample. This could not account for the 16.5 wt.% difference between the amorphous content in the original GP.-MTs and the GP. + 5% FAc. This indicated that the FAc was facilitating the reactivity and geopolymerization of the raw MTs. This was further corroborated by the decrease in the crystalline phases of the GP. + x% FAc samples compared to the original GP.-MTs sample and supported the FTIR observations of disappearing absorption bands corresponding to aluminosilicate phases in the raw materials. As FAc content increased in the co-geopolymerized samples, there was a decrease in amorphous content contribution from the MTs and a variable effect on the overall amorphous content, with the GP + 10% FAc sample showing the highest total amorphous content, followed by the GP + 20% FAc sample.

Zhu et al. [38] reported a similar observation in the production of alkali activated metakaolin/slag pastes, where the substitution of metakaolin (with very low calcium content) by slag (with high calcium content) increased the reactivity of precursors, contributing to a higher reaction rate and accelerated the geopolymerization process. Similarly, in the present case, during the dissolution of the FAc precursor, more calcium species were transferred into the solution; and the presence of these additional species increased the alkaline activation capability (OH^−^) because they locally raised the pH, which in turn increased dissolution of the local aluminosilicate particles and, thus, increased the extent of the inorganic polymeric structure [39].

**Figure 8:**
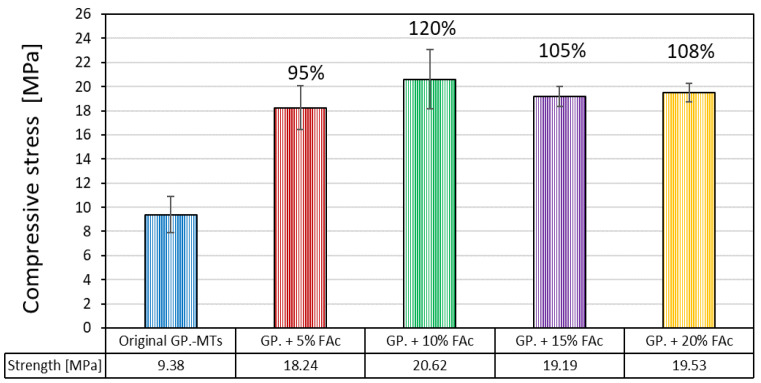
Comparative compressive strength of the original GP.-MTs with the co-geopolymerized systems at four different concentrations of FAc.

Uniaxial compression tests of the geopolymer specimens were performed after seven days of curing. The compressive strength for the co-geopolymerized specimens with FAc at four different concentrations of 5, 10, 15 and 20 wt.% of FAc increased for all the samples as is possible to observe in Figure 8. The specimens with 10 wt.% FAc showed the highest compressive strength of 20.62 MPa, which corresponded to an increase of 120% in comparison with the original GP.-MTs system. Similarly, the co-geopolymerized system with 20 wt.% FAc achieved a compressive strength of 19.53 MPa that corresponded with an increase of 108%. These two samples also had the highest total amorphous content indicating that as the amorphous content increased in the final co-geopolymer systems a direct improvement in their compressive strength values was obtained, which further supported the synergistic effect of the FAc on the MTs reactivity and co-geopolymerization, which was associated with their improved strength [27,42].

## 4. Conclusions

This study presented the results and analyses conducted to investigate the effect of the FAc in the co-geopolymerization process of low-reactive gold MTs. Characterizations of the final specimens at four different FAc/MTs ratios, based on uniaxial compressive tests, FTIR, and QXRD, were carried out to understand the interaction between both precursors during the inorganic co-geopolymerization process of these blended systems. From the results obtained, the following conclusions can be drawn:

(1) Based on the FTIR and QXRD results, it was found that both raw materials (raw MTs and raw FAc) could contribute to the additional amorphous phases with the presence of low quantity formed C-A-S-H crystalline phase in some of the generated specimens through the dissolution of their original aluminosilicate phases during alkali activation.
(2) The FTIR and QXRD results also indicated an increase in the dissolution of the gold MTs (low calcium content) by the addition of the FAc (rich in calcium) into the blended systems with a maximum value of formed amorphous content reported for the system with 10 wt.% of FAc. This suggests that the dissolution of FAc contributes to a major quantity of calcium species being able to react during the co-geopolymerization step and dissolving to a major degree the MTs’ aluminosilicates phases, such as the muscovite, albite, and even quartz.
(3) The final compressive strength of all co-geopolymerized systems based on gold MTs, and mixed with FAc at different concentrations, increased by 95–120% in comparison to the original MTs geopolymer system. The higher strength value was obtained by the blended system with 10 wt.%. FAc, which also showed the highest production of amorphous content after the co-geopolymerization step. This suggests that the improvement of the specimens’ strength is directly correlated to the increase in the amorphous phase production during the co-geopolymerization of these raw materials.

This work highlights the potential of improving the reactivity of low-reactive geopolymer precursors, such as low reactive gold MTs, through the incorporation of additional high-calcium supplementary materials.

## Figures and Tables

**Table 1:** XRD phases identified and their associated PDF codes.

Phase	Chemical Formula	PDF Code
Albite	Na(AlSi_3_O_8_)	04-017-1022
Anhydrite	(CaSO_4_)	01-074-2421
C-A-S-H	Ca_3_Al(Al_3_SiO_10_)(OH)_2_	00-001-1079
Gehlenite	Ca_2_Al_2_SiO_7_	01-089-6887
Grossular	Ca₃Al₂(SiO₄)₃	04-013-2106
Magnetite	Fe_2_O_3_	01-075-0449
Muscovite	KAl_2_(FOH)_2_ or (KF)_2_(Al_2_O_3_)_3_(-SiO_2_)_6_	00-001-1098
N-A-S-H	Na_17_._6_(Al_16_Si_56_O_144_)(H_2_O)_38_._4_	04-017-1022
Quartz	SiO_2_	01-077-8621
Synthetic Diamond	C	01-079-6061

**Table 2:** QXRD analyses of the raw materials (MTs and FAc), their own geopolymers, and the co-geopolymers of the MTs with different FAc content. Only phases contributing ≥ 0.5 wt.% of the sample are reported.

Phase	Raw MTs Wt.%	Original GP.-MTs Wt.%	Raw FAc Wt.%	FAc-GP. Wt.%	GP. + 5% FAc Wt.%	GP. + 10% FAc Wt.%	GP. + 15% FAc Wt.%	GP. + 20% FAc Wt.%
Muscovite	9.3	6.5	-	-	4.8	2.4	6.0	5.1
Quartz	51.1	38.9	5.4	1.6	29.3	25.4	27.6	24.6
Gehlenite	-	-	13.1	1.3	-	-	-	-
Albite	12.5	9.2	-	-	5.2	6.6	5.7	5.4
Grossular	-	-	9.6	1.7	-	-	-	-
Anhydrite	-	-	0.8	-	-	-	-	-
Magnetite	0.7	-	-	-	-	-	-	-
Zeolite	-	1.2	-	-	-	0.7	-	0.7
Calcium Aluminum Silicate Hydrate (C-A-S-H)	0.8	0.7	-	-	-	-	1	0.5
Sodium Aluminum Silicate Hydrate (N-A-S-H)	1.9	-	-	-	-	-	-	-
% of Amorphous	23.2	43.1	70.9	95.3	59.6	63.9	58.6	62.9

## Data Availability

Not applicable.
